# Open to MOOCs? Evidence of their impact on labour market outcomes

**DOI:** 10.1016/j.compedu.2021.104289

**Published:** 2021-11

**Authors:** Jonatan Castaño-Muñoz, Margarida Rodrigues

**Affiliations:** a European Commission, Joint Research Centre, Spain; b Fundação José Neves, Portugal

**Keywords:** E-learning, Lifelong learning, MOOCs, Employment

## Abstract

This paper investigates the effect that participation in massive open online courses (MOOCs) ecosystem can have on workers' labour market outcomes, mainly employment retaining. We use primary data collected between 2015 and 2017 and obtained from two surveys (pre-course and follow-up questionnaires) sent to participants in six MOOCs. The treatment group is composed of individuals participating in MOOCs in 2015 and the control group is composed of individuals who enrolled in MOOCs for the first time in 2017. Using a differences-in-differences approach, we find that participation in MOOCs can improve workers’ employment retaining but not their wages. The positive effect on employment retaining is homogeneous in different geographical and economic areas and tends to be higher for individuals who have participated in more MOOCs in the past.

## Highlights

• Participation in MOOCs is growing and switching towards professional development.
• The paper investigates employment retaining returns to participation in MOOCs ecosystem.
• The results show that participation in MOOCs improves employment retaining but not their wages.
• Heterogeneous effects are explored.
• Results can inform policies on the use of elearning and MOOCs to upscale training.

## 1 Introduction

The use of online educational innovations has become more common in recent years, and it is hoped that they will enhance human capital and decrease inequalities (Acemoglu, Laibson, & List, 2014; Hoxby, 2017). Among these educational innovations, massive open online courses (MOOCs) have gained notoriety in the educational landscape.^1^ These are online courses that have virtually unlimited enrolment capacity and effectively no enrolment requirements other than access to the internet. They are generally offered free of charge to participants, although some extra services, such as issuing certificates, may need to be paid for.

The first MOOCs were launched in 2008 and became ‘mainstream’ educational tools in 2012 (Pappano, 2012). In just a few years the expansion of MOOCs has been extraordinary: in the three first years of existence, between 2012 and 2015, more than 25 million people worldwide enrolled in MOOCs (Zhenghao et al., 2015). By early 2016, more than 4000 MOOCs were available (OECD, 2016) and in 2020 there were 16,300 courses that reached 180 million MOOC learners. 2020 was a remarkable year for MOOCs as one third of the learners that ever registered on a MOOC platform joined in that year.^2^ This figure reflects the extraordinary increment of enrolments associated to the COVID-19 crisis, which in some cases was 3 or 4 times higher than in similar periods of time of the previous year.^3^ The reasons for this unprecedented raise in enrolments are twofold. On the one hand, the higher interest in online learning linked to its massive use during the COVID-19 crisis (Bacher-Hicks, Goodman, & Mulhern, 2021; CEDEFOP, 2020) and, on the other hand, the fact that the need for upskilling and re-skilling workforce can be exacerbated as a part of the response to the economic crisis generated by the COVID-19 (Borio, 2020, Palomino et al., 2020). In this context and with such a large number of providers investing in MOOCs and individuals participating in them, it is both timely and relevant to study the impact of MOOC-based learning.

MOOCs are often discussed as a possible tool for scaling-up not only education, but also the provision of job-related training. OECD (2016) claims that integrating MOOCs into professional development is the most promising use for these educational instruments in the future. Compared with traditional learners, adult and employed individuals have different needs and expectations (Schuetze & Slowey, 2002) and value the flexible and self-directed learning opportunities that MOOCs can offer (Carnoy, Jarillo, Castaño-Muñoz, Duart-Montoliu, & Sancho-Vinuesa, 2012, Falconer et al., 2013). MOOCs providers are aware of this and are gradually offering more professional development content, rather than purely academic content (Reich & Ruipérez-Valiente, 2019). Thus, MOOCs are progressively becoming an alternative or complement to traditional lifelong learning options, such as public programmes, on-the-job-training or formal accredited courses.

Many higher education institutions are offering MOOCs to extend their traditional mission and offer short and flexible lifelong learning opportunities in non-degree programmes (European University Association, 2018) which are often related to professional development. In addition, research shows that MOOCs do not replace traditional higher education but are rather a marketing tool for increasing enrolments in traditional education (Jacqmin, 2019). Moreover, a recent study shows that the offer of a cheaper online master on computer science does not affect the demand of the same offer in the traditional format but expands the total demand (Goodman, Melkers, & Pallais, 2019) and MOOCs can play a similar role.

The relationship between MOOCs and labour market outcomes is an almost uncharted research area. However, it is relevant to explore it since MOOC characteristics differ to traditional types of adult learning, such as on-the-job training and formal higher education. The substantial differentiating characteristics of MOOCs may yield different returns. In addition to their above-mentioned specificities, MOOCs have a unique design in terms of instruction and interaction (Margaryan, Bianco, & Littlejohn, 2015) and lead to informal types of certificates and credentials, which may be less valued differently by employers than, for instance, on-the-job training (Egloffstein & Ifenthaler, 2017; Hamori, 2019; Rivas, Baker, & Evans, 2020; Rosendale, 2017). On the other hand, since participation in MOOCs is mainly an individual and intrinsically-motivated decision, it probably conveys a stronger signal to potential employers than that of on-the-job training (Radford et al., 2014). In addition, workers can be more aware of their own development needs than employers and may be in a better position to select adequate and effective courses. The open and flexible nature of MOOCs may facilitate this process.

From an education economics perspective, both human capital and signalling theories (Becker, 1964; Spence, 1973) predict a positive impact of MOOCs (when they are related to work) on labour market outcomes because participants acquire new or refresh existing job-related skills/knowledge and/or signal relevant characteristics to employers, such as perseverance, commitment, intrinsic motivation or interest in learning about novelties in their fields.

To the best of our knowledge, to date, the only study to have examined the relationship between participation in MOOCs and participants’ labour market outcomes is by Zhenghao et al. (2015). They present the results from a follow-up survey sent to participants in MOOCs offered on the Coursera platform that point to some labour market benefits of taking MOOCs. However, their analysis is purely descriptive and relies on the subjective evaluations of participants. It is an open question whether or not such reported benefits unfold in econometric analyses that use more objective labour market outcomes. This paper addresses this gap and is the first to evaluate the impact of participation in MOOCs on labour market outcomes using a control group and a quasi-experimental design. Our study focused on employed individuals since a substantial proportion of MOOC learners are employed adults and, in line with previous literature on MOOCs, our analysis is focused on individuals who have completed higher education.^4^

Our main research question regards how workers' participation in MOOCs ecosystem can impact their employment over time. We also give suggestive evidence on the impacts that MOOCs have on wages and on the probability that participants in MOOCs will change either their employer or the tasks they perform within their current firm. To address these questions, we took advantage of a unique primary data collection that surveyed participants in six MOOCs developed by Spanish universities and business schools in two specific fields very related to job-market skills: ‘Business Intelligence’ and ‘Communication and Marketing’. The data were collected between 2015 and 2017. Individuals who participated in selected MOOCs were surveyed first in 2015, before the MOOC took place, and then again two years later, in 2017. These individuals formed the treatment group. The control group was made up of individuals who enrolled in the selected MOOCs for the first time in 2017. They answered the pre-course questionnaire during the first half of 2017, and they were also asked to report retrospectively their 2015 labour market outcomes. To identify the effects of MOOCs we used a differences-in-differences approach in our analysis.

The results of our study show that participating in MOOCs ecosystem increases the workers' probability to still be employed two years later. When focusing on individuals who were employed at both points of time in the study, we find suggestive evidence that MOOCs have no impact on wages but increase the likelihood of workers continuing to work at the same firm and performing the same job. We conclude that MOOCs can be a good professional development instrument, even though this statement cannot be generalised to all individuals and topics, because we focus on MOOCs covering specific subjects and individuals participating actively in them. In addition, we go beyond estimating the effect of participating in MOOCs on the probability of workers to keep employed and explore heterogeneous effects in two dimensions that are particularly relevant to MOOCs. For instance, given the capacity of MOOCs to deliver *trans*-national learning, we study whether the employment return to participation in MOOCs differs with the geographical residence of the learner. We show that MOOCs developed in Spain impact positively on employment retaining elsewhere, at least in Latin America and Caribbean countries, supporting the idea that these can be ‘travel-well’ courses that have returns in other geographical, economic and labour market contexts. Another interesting heterogeneous effect relates to the number of MOOCs that individuals participate in. There has been some debate about whether or not these typically short-duration courses provide enough human capital to have an impact on labour market outcomes. However, the short duration of courses also enables more flexible and individualised learning approaches, whereby individuals can determine their own learning path by combining different MOOCs according to their specific needs. We show that individuals who have participated in more MOOCs in the past tend to have a higher probability to remain employed.

With these results, this paper contributes in several ways to at least three strands of the literature. *First*, it expands the literature on the effectiveness of online learning. This literature has mainly focused on analysing the effect of the delivery mode of online higher education degrees (Bettinger, Fox, Loeb, & Taylor, 2017; Escueta, Quan, Nicknow, & Oreopoulos, 2017). However, analyses of the impact of online higher education on labour market outcomes are scarce. Moreover, they tend to focus mainly on wage effects and generally find null or very limited effects in specific groups (e.g. Castaño-Muñoz, Carnoy, & Duart, 2016, Hoxby, 2017). In general, compared with those undertaking face-to-face degrees, online students (of online degrees and MOOCs) are older, more likely to be working already and receive higher wages. Thus, typical online learners have little room for improving wages and the analysis of outcomes related to job retaining can be more pertinent for this population. This paper contributes to the literature by examining a more appropriated outcome in the context of a special type of online course (MOOCs) that is shorter in nature than online degrees, hence providing more evidence on the value of online learning in the labour market. This last aspect is particularly important due to the impact of COVID-19 in the labour market. According to the OECD, 20 million jobs were destroyed in OECD countries and reskilling is urgent to employment recovery (OECD, 2021).

*Second,* the paper contributes to the growing body of literature that looks at the participants in MOOCs, which has, to date, been mostly limited to analysis of their profiles and motivations and has shown the difficulties that MOOCs are facing to fulfil their initial promise to democratise education (Lambert, 2020). It has been shown that participants in MOOCs are mainly over 30 years old and tend to be highly educated. Additionally, participation and success in MOOCs has been shown to require specific skills, such as organisational skills (Banerjee & Duflo, 2014), self-regulated learning skills (Littlejohn, Hood, Milligan, & Mustain, 2016) and digital skills (Castaño-Muñoz, Kreijns, Kalz, & Punie, 2017). MOOC-based learners report having several types of motivations to take part in this type of learning: job-related motivations and career benefits clearly play a role (Garrido et al., 2016; Kizilcec & Schneider, 2015; Zhenghao et al., 2015), along with intrinsic motivation, personal interest and enrichment (Barak, Watted, & Haick, 2016; Kizilcec & Schneider, 2015). There is rather less information available on employers' and recruiters' views of MOOCs, but descriptive evidence suggests that they value employees’ participation in MOOCs and see them at least as a valuable complement to other types of training (Garrido et al., 2016; Radford et al., 2014). Regarding the labour market returns to MOOC-based learning, a follow-up survey of participants in MOOCs offered by Coursera (Zhenghao et al., 2015) revealed that 85 % of the respondents reported intangible benefits (such as improved skills for their job or better opportunities in the labour market) and 33 % reported tangible benefits (such as wage increases or getting a new job).

*Third,* this paper contributes to the literature on adult education and training in two ways. While this literature has to date focused largely on traditional types of adult learning, such as on-the-job training and formal education, the substantial differentiating characteristics of MOOCs may yield different returns. MOOCs, in addition to their above-mentioned specificities, have a unique design in terms of instruction and interaction^5^ and lead to informal types of certificates, which may be less valued by employers than, for instance, on-the-job training. On the other hand, they probably convey a stronger signal than that of on-the-job training. In addition, we also contribute to the small literature on the employment effects of adult learning. While most of the literature focuses on wage effects,^6^ the impact on employment retaining should not be neglected, especially in times of vulnerable job security (Hansson, 2008). Gaining a qualification during adulthood is found to promote the transition from non-employment to employment (see, for example, Dorset, Liu, & Weale, 2010; Hällsten, 2012; Jenkins Vignoles, Wolf & Galindo-Rueda, 2003). For workers, on-the-job training is associated with improved job security and job retention (Hansson, 2008), i.e., with the probability that workers will keep their employment status. Picchio and van Ours (2013) find that firm-provided training in the Netherlands decreases the probability of becoming non-employed by around 3.5 percentage points. This positive relationship between training and employment retaining is confirmed by our analyses.

The paper proceeds as follows. The next section presents the data and sample used. Section 3 describes the empirical methodology and section 4 presents the estimation results and robustness checks. In section 5 we discuss our results and put them in perspective with the related literature. In section 6 we present the conclusions and discuss the policy implications of our results.

## 2 Data

### 2.1 Data collection and MOOCs surveyed

Our study uses data from a primary data collection on MOOC takers, carried out in the context of a broader research project of the European Commission's Joint Research Centre (JRC): MOOCKnowledge.^7^ The research team agreed with several MOOC providers to send standardised questionnaires to individuals who had enrolled in their MOOCs (hereafter ‘surveyed MOOCs’). The questionnaires follow the theoretical framework of the MOOCKnowledge project and were validated by experts and tested in pilots previous to the data collection (see Kalz et al., 2015). Most of the variables used in the analysis are single items. When scales are used (see point 4.4), we present evidence on their reliability (Cronbach's alpha) and construct validity (factor analysis).

There were two questionnaires. The *pre-course questionnaire (Pre-Q)* was sent to participants after their enrolment in the MOOC and was completed by them (on a voluntary basis) before they started the course. It aimed at collecting data on individual characteristics, labour market information, participation in lifelong learning and motivations to take the MOOC. The link to complete the *Pre-Q* was available on the MOOC platform once it started. Our sample is therefore made up of active learners who, at least, logged into the MOOC environment and engaged with the course activities^8^ (Evans, Baker, & Dee, 2016; Koller, Ng, Do, & Chen, 2013; Reich, 2014). The *follow-up questionnaire (Fup-Q)* was sent to participants two years after they enrolled in the surveyed MOOCs and collected information on labour market variables and the self-reported impact of MOOCs.

Data collection started in 2015 when the *Pre-Q* was sent to students who enrolled the surveyed MOOCs starting that year. The *Fup-Q* was sent during the first half of 2017 to the individuals who have answered the *Pre-Q*. In the first half of 2017, the *Pre-Q* was sent to individuals enrolling in surveyed MOOCs starting in 2017 on the same or similar topics as the 2015 surveyed MOOCs.

The main analysis presented in this paper uses data from six MOOCs^9^: two took place in 2015 (data available for both *Pre-Q* and *Fup-Q* – treatment group); and four started in 2017 (data available for the *Pre-Q* only – control group). The broad fields of the surveyed MOOCs were ‘Business Intelligence’ and ‘Communication and Marketing’. The former deals with data analysis and big data, while the second is concerned with communication skills. The ‘Business Intelligence’ MOOC seems to provide more specific skills than the ‘Communication and Marketing’ MOOC. These MOOCs were developed by Spanish universities and business schools and were offered in the Spanish language. On average, each MOOC lasted for a total of 15 h, spread over two to five weeks, with a weekly workload varying between 4 and 5 h^10^. The number of enrolments in the MOOCs ranged between 700 and 18,000^11^

### 2.2 Treatment group

The treatment group comprised individuals who enrolled in the surveyed MOOCs in 2015 and who answered both the *Pre-Q* in 2015 and the *Fup-Q* in 2017. This gave a total of 138 individuals, of whom 80 enrolled in the ‘Communication and Marketing’ MOOC and 58 in the ‘Business Intelligence’ MOOC. Despite the considerable sample attrition, the treated sample that originally answered the *Pre-Q* is, in general, statistically similar to the sample that answered the *Fup-Q*.^12^

Table 1 shows that the individuals in the treatment group were, on average, 45 years old and 90 % had completed higher education, and that most came from Spain or from Latin American or Caribbean countries. In addition, on average, they had already participated in six MOOCs before taking part in the surveyed MOOC.

**Table 1:** Characteristics of treatment and control groups at the beginning of the MOOC.

	All MOOCs			Communication and marketing			Business intelligence		
	Treatment	Control	Test	Treatment	Control	Test	Treatment	Control	Test
Number of observations	138	208		80	82		58	126	
Average age (years)	44.5	38.8	***	45.8	35.7	***	42.6	40.8	
Gender (% male)	53.6	49.0		40.0	30.5		72.4	61.1	
Area of residence (%)									
Spain	63.0	53.3	*	67.5	48.8	**	56.9	56.4	
EU (non-Spain)	1.45	2.4		1.3	4.9		1.7	0.79	
Latin America and Caribbean	35.5	43.3		31.3	45.1	*	41.4	42.1	
Other	0.0	0.96		0.0	1.2		0.0	0.79	
Education level (% with HE)	89.1	93.3		87.5	95.1	*	91.4	92.1	
Area MOOC (%)									
Business Intelligence	42.0	60.6	***	–	–		–	–	
Communication and Marketing	57.9	39.4		–	–		–	–	
MOOCs enrolled in the past	6.2	0		7.2	0		4.7	0	
High belief in labour market benefits (%)	81.9	81.9		80.0	91.0	**	84.5	76.0	
Completed the surveyed MOOC (%)	78.3	–		78.8	–		77.6	–	

*Notes*. The table presents descriptive statistics for the baseline characteristics of treatment and control groups, for the pooled sample and for each MOOC field separately. ‘Test’ columns indicate whether the difference between treatment and control groups is statistically different from zero. *p < 0 0.1, **p < 0.05, ***p < 0.01. HE, Higher education.

The profiles of individuals enrolling in the different MOOCs varied: compared with the participants in the ‘Communication and Marketing’ MOOC, those in ‘Business Intelligence’ were more likely to be men, younger and to have participated in fewer MOOCs in the past. Around 80 % of the participants in both MOOCs fields self-reported that they had completed the course. Contrasting this number with the typically high drop-out rate for MOOCs, it is clear that the sample of treated individuals comprises particularly motivated learners and not the average profile of individuals enrolled in MOOCs.

### 2.3 Control group

Ideally, the impact of the MOOCs would be estimated by conducting a randomised control trial in which participants are randomly allocated to the treatment or control group. In the absence of such experimental options, the choice of the control group and of the identification strategy is of crucial importance to estimate the effect of MOOCs on labour market outcomes (Oosterbeek, 2013). One potential set of controls would be individuals following the same employment path as the treatment group but not enrolling in MOOCs in 2015. Unfortunately, we do not have any information on pre-2015 labour market outcomes. In addition, such a control group would completely overlook the issue of selection into training. Leuven and Oosterbeek (2008) suggest using a control group that is similar to the treatment group in terms of observed individual characteristics and the characteristics of the training. They narrow down the control group to individuals who planned to participate in training but did not because of a random circumstance. In our case, individuals were only able to answer the pre-questionnaire in the MOOC platform. We were therefore unable to follow such a strategy.

Our control group is composed of individuals who enrolled in surveyed MOOCs in 2017 and answered the 2017 *Pre-Q*. Apart from asking questions about 2017 demographic characteristics and labour market information, this *Pre-Q* also included questions about employment status and wage levels two years before. In the absence of standard longitudinal data for the control group, we relied on the participants’ retrospectively self-reported employment status and wages as outcomes for the baseline period (2015). The control group was composed of individuals who reported being employed in 2015. In order to have a clear definition of treatment, we restricted the control group to those for whom the surveyed MOOC (in 2017) was the first they had ever participated in, so as to avoid their participation in MOOCs between 2015 and 2017.^13^

The control group comprised total of 208 individuals, of whom 126 had enrolled in the ‘Business Intelligence’ MOOC and 82 in the ‘Communication and Marketing’ one. In the case of the ‘Business Intelligence’ MOOC, the 2015 and 2017 courses were different editions of the very same MOOC (first and third edition, respectively). Therefore, all the MOOC characteristics were the same – provider, duration, content, etc. For the ‘Communication and Marketing’ field, the MOOC content was similar but not exactly the same. Hence, the ‘Communication and Marketing’ MOOCs surveyed in 2015 and 2017 may not share the exact same characteristics.^14^

*A priori*, this is a particularly valid control group since the individuals included in it have also self-selected to undergo further training in the same study areas, have chosen a MOOC to do so and have started learning activities. The difference is that this group opted into the same treatment at a different point in time (Hoxby, 2017); the assumption is therefore that the timing of enrolment in MOOCs is random. Indeed, it is very likely that the control group shares characteristics with the individuals in the treatment group, notably unobservable characteristics that are likely to be systematically related to participation in MOOCs and labour market outcomes, such as motivation and ability. As for the observable variables, a comparison between the treatment and the control group (Table 1) reveals no major differences. In the case of the general sample and those enrolling in ‘Communication and Marketing’ MOOCs, individuals in the treatment group are, on average, older and more likely to come from Spain. Importantly, there are no statistically significant differences in the observable characteristics examined between the treatment and control groups enrolling in the ‘Business Intelligence’ MOOCs.

## 3 Empirical methodology

The empirical literature on the returns to adult learning has used several approaches to deal with endogeneity bias (Oosterbeek, 2013). One of them is the Heckman-type selection correction, but it has proved difficult to find sources of exogenous variation. A second, and more widely used, approach is a fixed-effects/differences-in-differences setting. Finally, Leuven and Oosterbeek (2008) and, later, Guerlitz (2011) propose a simple ‘ordinary least squares’ (OLS) approach that is able to mimic an experimental setting by making use of a comparison group of individuals who planned to take training but ended up not doing so because of random events.

Given the specificities of our control group and the fact that we have data on the main labour market outcomes for both the treatment and control groups in 2015 and 2017, our approach combines, to some extent, the last two approaches: we apply a differences-in-differences strategy using individuals who also enrolled in MOOCs, but at a different point in time, as a control group.

In the absence of time-varying covariates – as in our data – the impact of MOOCs is obtained by directly comparing the before-after differences between the treatment and control groups. The standard differences-in-differences regression is useful for inference purposes. The estimated model is as follows:

(1) $$Y_{i,t}=\alpha +\beta D_{i}*time+\gamma_{1}D_{i}+\gamma_{2}time+\delta X_{i}+\varepsilon_{i,t}$$

where $Y_{i,t}$ is the employment indicator of individual i at time t (or the wages), $D_{i}$ is the treatment dummy, *time* is a dummy variable taking the value of 1 in 2017 and $X_{i}$ is the set of pre-enrolment characteristics directly linked to job market (age, gender, education level, residential area and MOOC field).^15^ Given the two-period setting, this specification is equivalent to a fixed-effects model.

The differences-in-differences estimation allows for individual-specific and time-specific factors, ruling out the threat of biased estimates driven by time-invariant factors (observed or unobserved, such as motivation, innate ability, personality traits, propensity to perform well in the job, propensity to follow lifelong learning etc.).

The main assumption is that individuals in both the treatment and control groups were following common trends in the labour market outcomes variables. Unfortunately, we do not have this type of information prior to 2015 and are not able to test this assumption. However, the fact that we limit our analysis to employed individuals in 2015 who are active MOOC learners deals with this issue. While the existence of time-varying heterogeneity cannot be completely ruled out, we stress that the control group is a particularly valid one for the reasons presented above. Therefore, selection bias is certainly much lower than if we had used a generalised control group that may never have participated or planned to participate in lifelong learning.

## 4 Results

We present results for the two MOOC fields together and also separately for the MOOC fields of ‘Business Intelligence’ and ‘Communication and Marketing’, thus assessing possible heterogeneous effects across MOOC fields. This is relevant since the former field provides more specific/technical skills and the latter more soft/generic skills. The results for the ‘Business Intelligence’ MOOC are particularly relevant for two reasons. First, the 2015 and 2017 MOOCs are completely comparable (the 2017 MOOC being the third edition of the 2015 course). Second, the treatment and control groups have balanced observed characteristics (see Table 1).

### 4.1 How does participation in MOOCs impact workers’ employment retaining?

Table 2 shows the proportion of employed individuals in the treatment and control groups, both in 2015 and in 2017. In 2015 all individuals were in employment. In 2017, among the treatment group (i.e. those who enrolled in the surveyed MOOCs in 2015), 6.5 % were not in employment, accounted for by a reduction in employment of 5.2 % among the group enrolled on the ‘Business Intelligence’ MOOCs and of 7.5 % in the ‘Communication and Marketing’ MOOC group. This change in employment status could have occurred even in the absence of participation in MOOCs and, therefore, must be contrasted with the evolution of the employment rates in the control group. This group experienced an overall employment rate reduction of 13.9 percentage points, with a slightly greater fall in employment among those enrolling in the ‘Communication and Marketing’ MOOCs (−14.6 percentage points).

**Table 2:** –Share of employed individuals in treatment and control groups.

		All MOOCs	Business intelligence	Communication and marketing
Treatment group	2015	100	100	100
	2017	93.5	94.8	92.5
	Difference 2017–2015	−6.5	−5.2	−7.5
Control group	2015	100	100	100
	2017	86.1	86.5	85.4
	Difference 2017–2015	−13.9	−13.5	−14.6

*Notes*. The table presents descriptive statistics for the proportion of employed individuals in the treatment and control groups in 2015 and 2017.

The differences-in-differences estimates, shown in Table 3, point to a positive and significant effect of participation in the MOOCs. For the overall sample, enrolling in MOOCs increases the probability of remaining employed in 2017 by 7.4 percentage points. The estimate is slightly higher for Business Intelligence MOOC learners (8.3 percentage points) and significant at a standard significance level. For the ‘Communication and Marketing’ MOOC learners, the estimate is marginally significant and amounts to 7.1 percentage points.

**Table 3:** Effect of participation in MOOCs on the probability of remaining in employment.

	All MOOCs	Business Intelligence	Communication and Marketing
β	0.074**	0.083*	0.071+
(s.e.)	(0.034)	(0.049)	(0.049)
*N*	346	184	162

*Notes*. The table presents the results from the differences-in-differences regression (1). The dependent variable is a dummy variable indicating employment. Individual control variables include age, gender, indicator for higher education degree and residential location. Robust standard errors in parenthesis. +p < 0.15; *p < 0.1; **p < 0.05; ***p < 0.01.

Given that the individuals in the treatment group have, on average, not only participated in the surveyed MOOC but also in around 6 MOOCs before it and 3 MOOCs after it (during the period between the surveyed MOOC and the follow-up questionnaire), these estimates may encompass the effect of the participation in several MOOCs. In fact, it may be that it is precisely the accumulation of skills from several MOOCs that gives an advantage in the probability of remaining in employment. If the estimate of 7.4 percentage points corresponds to the average total number of MOOCs the treatment group participated in (10), it means that, on average, each MOOC increases the probability of remaining in employment by 0.74 percentage points.

The estimates of the effect of MOOCs may be driven by particular groups of individuals in our sample, in which case we would be erroneously generalizing the benefits of MOOCs. For instance, our sample includes individuals from different geographic and economic contexts and with different levels of experience in MOOCs and it could be the case that the estimates only hold for one geography and for experienced learners. In order to have accurate conclusions, we explore heterogeneous effects by analysing whether the estimated effect holds in different national contexts and differs depending on how experienced individuals are with MOOCs. In both cases we run the differences-in-differences regression with two treatment variables and test for equality of their coefficients.

In Panel A of Table 4, we examine whether the effect of participating in MOOCs differs for individuals residing in Spain or elsewhere. In both MOOC fields and in the pooled sample, the estimates for the two locations are similar and not statistically different from each other. This finding indicates that MOOCs developed in Spain have a return on employment retaining in Europe but also outside Europe, which strongly supports the assertion that MOOCs may open up learning possibilities and develop relevant skills in different geographical and economic contexts. However, it should be highlighted that the non-Spanish participants live mainly in Latin America and, although the socio-economic context is different, the cultural proximity and language of instruction could explain this similarity in the estimates.

**Table 4:** Results for heterogeneous effects.

	All MOOCs	Business intelligence	Communication and marketing
PANEL A: Heterogeneity with respect to residential area			
Effect for Spanish residents	0.070*	0.074	0.072
(s.e.)	(0.039)	(0.061)	(0.055)
Effect for other individuals	0.080*	0.067	0.069
(s.e.)	(0.048)	(0.068)	(0.071)
Equality of coefficients: F.st. (p-value)	0.03 (0.8536)	0.06 (0.8021)	0.00 (0.9697)
N	346	184	162
PANEL B: Heterogeneity with respect to previous experience with MOOCs			
≤5 MOOCs in the past	0.074*	0.054	0.096+
(s.e.)	(0.041)	(0.058)	(0.061)
>5 MOOCs in the past	0.074+	0.135*	0.046
(s.e.)	(0.045)	(0.073)	(0.061)
Equality of coefficients: F.s.t (p-value)	0.00 (0.9905)	0.92 (0.3374)	0.51 (0.4774)
N	346	184	162

*Notes*. This table shows the results of a version of the differences-in-differences regression (equation (1)) for which there are two treatment groups. The dependent variable is a dummy variable indicating employment. Individual control variables include age, gender, indicator for higher education degree and residential location. Robust standard errors in parenthesis. +p < 0.15; *p < 0.1; **p < 0.05; **p < 0.01.

As regards previous experience with MOOCs, we divided the individuals in the treatment group into two subgroups: less experienced individuals (if they had participated in up to five MOOCs in the past) and more experienced individuals (if they had participated in more than five MOOCs in the past). Although Panel B of Table 4 reveals no statistical differences between the effects of less and more experienced MOOC takers, we find some differences between the two MOOC fields. The estimate is substantially higher for the ‘more experienced’ treatment group taking the ‘Business Intelligence’ MOOC. The reasons for this larger estimate for the ‘more experienced’ subgroup can be manifold. These individuals may have gained experience that allows them to use MOOCs more efficiently – for instance, they may be more familiar with the digital platforms used by MOOC providers or may be better at identifying the MOOCs that offer training in the skills they are in need of. Moreover, both signalling and human capital mechanisms could be at stake – participating in more MOOCs may provide a particularly strong and positive signal to employers and/or may be necessary to accumulate enough human capital to be valued by employers.

### 4.2 Robustness checks

We carry out numerous different robustness tests of our main estimates and present the results in Table 5.

**Table 5:** Results from robustness checks.

Panel A: Robustness with different control groups				
		All MOOCs	Business intelligence	Communication and marketing
(1) Impose Common Support	β	0.073**	0.082*	0.069
	(s.e.)	(0.034)	(0.049)	(0.050)
	*N*	344	183	160
(2) DID with matching	β	0.087**	0.089**	0.094
	(s.e.)	(0.041)	(0.045)	(0.082)
	*N*	346	184	162
(3) Low belief in labour market benefits	β	0.073+	–	–
	(s.e.)	(0.051)		
	*N*	174		
(4) Control group providing email	β	0.095**	0.107**	0.088+
	(s.e.)	(0.038)	(0.054)	(0.058)
	*N*	263	140	123
Panel B: Robustness with MOOCs targeting teachers				
MOOCs for teachers	β	0.020		
	(s.e.)	(0.024)		
	*N*	544		

*Notes*. The table presents results from several robustness checks, all of them obtained from the differences-in-differences regression (equation (1)). The dependent variable is always a dummy variable indicating employment and individual control variables include age, gender, indicator for higher education degree and residential location. The first row presents results when observations that lie outside the common support are dropped. The second row presents results of the differences-in-differences regression combined with matching on the baseline characteristics. In the third row we restrict the control group to those who do not have strong beliefs on the career opportunities expected from participating in the MOOC. The fourth row presents results using the subset of the control group that has provided an email address to be contacted in the future. In part B, we present the results from equation (1) using a different group of MOOCs that were targeted at teachers. Robust standard errors in parenthesis. +p < 0.15; *p < 0.1; **p < 0.05; ***p < 0.01.

*First*, we deal with the issue of common support (row 1). We assume that, for each individual in the treatment group, there is a comparable individual in the control group, but this may not be the case. Our estimates do not change qualitatively or substantially when the common support is imposed prior to the estimation of the effect of participation in MOOCs on the probability of remaining in employment^16^

*Second*, we run differences-in-differences regressions, matching treatment and control groups on pre-treatment characteristics (row 2). The result for the ‘Business Intelligence’ field is very similar to the original one, which is not surprising given that characteristics were already balanced across the groups before matching (see Table 1). For ‘Communication and Marketing’, the estimate increases but is less precisely estimated and hence not significant.

*Third*, we address the concern that some individuals in the control group may have enrolled in MOOCs in 2017 because they were unemployed but would not have done so otherwise. This issue is, to some extent, related to the Ashenfelter dip concept that is transversal to the literature on lifelong learning and according to which there can be a ‘dip’ in employment levels in the periods leading up to training. In principle, compared with other training types, this is less of an issue in MOOCs because of the relatively low workload and flexibility inherent in online platforms. Nevertheless, we provide two pieces of evidence that support the idea that our estimates are not severely affected by this possibility. First, in an attempt to isolate the individuals in the control group who would have enrolled in MOOCs regardless of their employment status, we disregard those who consider their participation in the MOOC as useful or extremely useful for their labour market chances. The estimate is very similar (row 3), but less precisely estimated because of the reduced number of observations. Second, we sent a short follow-up questionnaire to those enrolled in the surveyed MOOCs in 2017 (including also those who had previous experience in MOOCs). In this follow-up, we explicitly asked those who had reported being unemployed in the pre-questionnaire whether or not they would have taken the MOOC had they been working. It is reassuring to note that 80 % of them gave a positive answer.

*Additionally*, we address the concern that some individuals in the control group may not answer a follow-up questionnaire when given the chance, in which case individuals from the treatment group would be an even more selected group. Hence, we use a more conservative control group by restricting it to the individuals who granted permission to contact them in the future and provided their email addresses to that end. This reduction of 83 individuals in the control group delivers similar results, or even slightly higher estimates (row 4). We also used the response to the short follow-up questionnaire sent to the control group for this purpose. Again, limiting the control group to those who completed this follow-up questionnaire barely changes the results.^17^

*Finally*, to validate our approach we apply the core methodology to a different set of MOOCs where we do not expect to find a significant effect on employment status. We focus on MOOCs that were part of the same data collection but targeted at teachers – Panel B of Table 4. These ‘Teacher Training’ MOOCs are aimed at improving digital competences and teaching quality and, in principle, are used only by teachers. Since teaching is a particularly regulated profession, less vulnerable to business cycles than other professions, we anticipate a null estimate. It is reassuring to obtain a much smaller estimate for this MOOC field that, despite the larger number of observations, is far from statistically significance.

### 4.3 How does participation in MOOCs impact wages?

After having extensively analysed the impact of participation in MOOCs on the probability of remaining in employment, we now question whether MOOCs had an effect on the wages of individuals employed in both 2015 and 2017. We limit this analysis to European residents^18^ and present results only for the two MOOCs combined to guarantee a minimum number of observations. Even so, the correspondent number of observations is only 97 and we therefore interpret the results from this analysis with caution.

The information on wages is coded in 28 categories, each including a range of €3000, and we use them in a differences-in-differences (linear) regression. The estimate points to an estimate that is not statistically significant but also close to zero^19^: 0.093 (s.e. = 1.221).

### 4.4 Reconciling the employment retaining and wage effects

Our estimates point to a positive effect of participating in MOOCs on employment retaining, but there is evidence suggestive of a null effect on wages. These results are aligned with the subjective evaluation of the benefits of the surveyed MOOCs that was self-reported by the treatment group in the follow-up questionnaire.

The follow-up questionnaire contained a battery of questions aimed at uncovering the subjective usefulness of MOOCs. These questions can be divided in two different dimensions composed of four items each: the first dimension (1) relates to the perceived impact of MOOCs in acquiring skills and performing better in the current job; the second dimension (2) relates to getting a promotion or changing employer to secure a better job^20^. In order to assess which of the two types of benefits was perceived as more important by the participants in the MOOCs, we computed the item mean for each dimension and compared them. Workers considered participation in MOOCs to be more useful in dimension 1 (mean = 3.65), i.e. in increasing their skills and performing better in their current job rather than for dimension 2 (mean = 2.13), obtaining better job positions (t = −15.57, p < 0.0001). These results are consistent with our previous findings and apply to both the ‘Business Intelligence’ and ‘Communication and Marketing’ MOOCs^21^

These results suggest that one mechanism through which participation in MOOCs impacts employment retaining is through gaining occupation-, firm- or even task-specific skills that increase the likelihood of being employed in the same firm and/or performing the same job. In order to give an insight whether or not this mechanism is relevant, we looked at the probability of workers changing firm or functions. We dispose of such information only for the treatment group, which means that we cannot estimate directly the effect of participation in MOOCs on this outcome.

Alternatively, in an OLS setting controlling for several factors, we estimate the association between the number of MOOCs already taken and the probability of changing firm or function. We find suggestive evidence of a negative association (1.5 percentage points^22^) of participating in one extra MOOC and the likelihood of changing firm or functions. This implies that workers participating intensively in MOOCs may be searching for training in skills that they lack in their current job and that are occupation/job specific.

## 5 Discussion

The interest in how new forms of education and training enabled by digital technologies impact skills development and labour market outcomes is not new but has been accelerated by the COVID-19 crisis. Among these new digital educational tools, MOOCs are particularly relevant because of their rapid growth and the number of individuals enrolled. The fact that MOOCs are progressively becoming a tool for adult learning is pertinent in the light of the ever-increasing need for lifelong learning due to the social and technological changes taking place in contemporary societies.

We contribute to the literature with the first evidence on the effects of participating in MOOCs ecosystem on several labour market outcomes of workers, with a focus on the probability of remaining in employment.

Our findings indicate that workers participating in MOOCs ecosystem are significantly more likely to remain in employment after two years. This result is in line with literature on workers’ training that show positive effects on job retaining (Picchio & van Ours, 2013) and confirms that online courses can play a similar role, even if taken out of the firm context.

The positive effect shown here, can be explained by two factors. On the one hand, it can be an effect of the acquisition of the curriculum content of the analysed courses that is directly linked to labour market. On the other hand, it may also be that participation in MOOCs is useful to develop transversal skills that are valued in the job market. Literature has shown the relationship among employees’ participation in MOOCs and skills such as communication, planning, strategy, digital and technical competence, and innovation in companies (Karnouskos, 2017). If there were a causal relationship and these and other similar skills as teamwork, curiosity, self-regulation etc. are (intentionally or unintentionally) developed through MOOC participation, they could also partially explain the positive effect found in this paper.

Our estimates vary only slightly across the MOOC fields, but the ‘Business Intelligence’ MOOC is particularly relevant since the data on treatment and control group individuals arise from different editions of the same MOOC. In terms of heterogeneous effects, the estimates are similar for individuals living in the MOOC country of origin and elsewhere. This is encouraging since it supports the idea that MOOCs are ‘travel-well’ courses that can deliver *trans*-national learning opportunities. Moreover, the effect of MOOCs on employment retaining seems to be more pronounced for individuals who have already taken part in several MOOCs in the past, which could reveal non-linear learning gains, a more efficient selection and use of MOOCs by individuals for configuring specific training paths and/or a stronger signalling effect to employers.

As for other labour market outcomes, we find no effect on wages. This result contradicts literature on adult education and training that tend to show positive, although small, effects (Field, 2012; Oosterbeek, 2013), but the finding is aligned with the literature on online learning that tend to show null or very limited effects on wages (Castaño-Muñoz, Carnoy, & Duart, 2016, Hoxby, 2017). On the other hand, we do find suggestive evidence that MOOCs work to ‘shield’ the current job position of participants via the acquisition of job-specific competences, rather than acting as a springboard for obtaining better or new positions. The subjective workers' perception data corroborate these findings, since it was revealed that workers regard MOOCs as especially useful for increasing their skills and enabling better performance in their current job.

Overall, although the literature shows that MOOCs play a minor role in employers hiring and training decisions (Egloffstein & Ifenthaler, 2017; Hamori, 2019; Rosendale, 2017), our results indicate they MOOCs can be providing an alternative option for workers to develop useful skills and remain employed.

## 6 Conclusions

This paper estimates the effects of participating in MOOCs ecosystem on several labour market outcomes of workers, with a focus on the probability of remaining in employment. The general conclusion is that MOOCs are potentially a good professional development instrument for adult learners that can help individuals avoid spells of unemployment. However, this statement cannot be generalised and interpreted as the effect of an average MOOC taker for two reasons. First, our survey covered a limited number of MOOCs (focused on skills related to labour market). Second, our treatment group may not be representative of all MOOC participants, since it is particularly motivated and active learners who have participated recurrently in the MOOC ecosystem in the past.

Despite these limitations, our analysis provides support for the idea that MOOCs can offer new and useful learning opportunities, at least to highly motivated individuals. Owing to their open nature, MOOCs enlarge training possibilities and can be used as a scalable and flexible way to upskill and reskill workforce during and in the aftermath of the COVID-19 crisis.

This evidence on the labour market impact of MOOCs should be complemented with future research. First, it remains an open question whether or not MOOCs can improve the situations of individuals with poorer labor market careers. Second, it is also important to study the variables related to workers participation in MOOCs, including the role that employers may have and compare different settings: during free time or in working hours. In fact, employers can support the use of MOOCs without transferring all of the training responsibility to individuals. Finally, while labour market outcomes are an important part of the puzzle, it is not the only outcome of interest for MOOC learners or course providers and should not be the only criterion guiding policies and investment in this area. It would be therefore relevant to study the impact of MOOCs on other outcomes not covered in this paper.

The research questions raised here and their results will certainly animate policy-makers’ and practitioners’ discussions in the lifelong learning field. From a public perspective, and taking into account that MOOCs are costly for providers (Hollands & Tirthali, 2014), it can inform educational policy-makers on the adequacy of the direct or indirect (via public higher education institutions) use of public money in the creation of MOOCs.

## Author contributions

Jonatan Castaño Muñoz: Project administration, Resources, Conceptualization, Data curation, Formal analysis, Investigation, Methodology, Validation, Writing - original draft, Writing - review & editing Margarida Rodrigues: Conceptualization, Data curation, Formal analysis, Investigation, Methodology, Software, Validation, Writing - original draft, Writing - review & editing.

## Figures and Tables

**Table 6:** Information on the surveyed MOOCs.

	Code	T/C	Enrolment (N)	Country	Provider	Language	Duration	Content
Business Intelligence	MOOC B1 (1st edition)	T	18,268	Spain	Public University	Spanish	5 weeks	Data analysis and big data
							4 h x week	
	MOOC B1 (3rd edition)	C	9769	Spain	Public University	Spanish	5 weeks	Data analysis and big data
							4 h x week	
Communication and marketing	MOOC C1	T	16,737	Spain	Private university	Spanish	5 weeks	Public speaking and face-to-face communication
							4 h x week	
	MOOC C2	C	1570	Spain	Business school	Spanish	2 weeks	Online communication and social media
							5 h x week	
	MOOC C3	C	712	Spain	Business school	Spanish	2 weeks	Online communication and marketing
							5 h x week	
	MOOC C4	C	1383	Spain	Business school	Spanish	2 weeks	Web positioning strategies.
							5 h x week	

## Footnotes

1 See OECD (2016) for an overview of trends and future perspectives of MOOCs.

2 See https://www.classcentral.com/report/mooc-stats-2020/.

3 See: https://theconversation.com/massive-online-open-courses-see-exponential-growth-during-covid-19-pandemic-141859 and https://www.classcentral.com/report/mooc-stats-pandemic/for an overview.

4 See, for example, Castaño-Muñoz, Kreijns, Kalz & Punie (2017); Christensen et al. (2013); Glass, Shiokawa-Baklan, and Saltarelli (2016); Hansen and Reich (2015); Ho, Chuang, Reich, Coleman, Whitehill, Northcutt, et al. (2015); and OECD (2016).

5 Research has pointed to some shortcomings in the quality of large-scale students' settings (e.g. Margaryan et al., 2015).

6 While this evidence is mixed, there is the overall consensus that, once selection into training/education is accounted for, the wage effects are modest (see, for example, reviews by Field [2012] and Oosterbeek [2013]). In a meta-analysis, Haerlemann & Borghans (2012) find that the average reported wage effect of on-the-job training is 2.6 % per course. The effects are rather heterogeneous and studies using panel data tend to systematically report lower results.

7 The project aims at achieving a better understanding of the profile of MOOC takers and of the impact of MOOCs as a type of lifelong learning. For these purposes, the JRC agreed with several MOOC providers to send self-developed questionnaires to MOOC takers.

8 Despite the large number of enrolments in MOOCs in general, the drop-out rate is very high (Breslow et al., 2013; Jordan, 2014; Koller et al., 2013; Perna, Ruby, & Boruch, 2014), which probably reflects the trial and error process followed by participants, driven by the very small cost of enrolling on such courses (Hoxby, 2014).

9 The data collection also included six MOOCs targeted at teachers who provide teacher training in ICT and blended learning (two for 2015 and four for 2017). We have excluded these from our main analysis owing to the specificities of teachers' labour markets and professional development systems. These data will be used later in the paper in a placebo analysis (see section 4.2).

10 See the appendix for detailed information on the surveyed MOOCs.

11 The figures on enrolment do not reflect the number of active participants as a substantial proportion of enrolled individuals never access the MOOC platform. These numbers cannot, therefore, be used for estimating the response rates to our surveys. In the case of the 2015 (2017) Business Intelligence MOOC, we know that only 35 % (22.3 %) of those enrolled completed at least the MOOC's first module. These are (roughly) the individuals who could have answered our *Pre-Q*. Using these figures we get a response rate of 9.6 % (first edition) and 16.7 % (third edition) to our *Pre-Q* for this specific MOOC. Unfortunately, we do not have information that allows us to estimate the response rate for the Communication and Marketing MOOC.

12 Of the 767 individuals who answered the *Pre-Q* in 2015, 138 also answered the *Fup-Q* in 2017 (response rate of 18 %). The two groups are similar in terms of residential area, gender, education level, area of the MOOC, self-reported digital competence and beliefs regarding the labour market benefits of taking the MOOC. Compared with those who did not answer the follow-up questionnaire, those who did were, on average, four years older and had participated in more MOOCs in the past (average difference of 1.5). The comparison of these two groups is available from the authors upon request.

13 Around 30 % of individuals answering the 2017 *Pre-Q* had no previous experience of MOOCs.

14 Often, MOOCs take place only once and, therefore, it was not possible to collect data on individuals taking the same MOOC later on.

15 The variables contained in *X* are fixed over time and, therefore, affect the estimation not of the coefficient of interest but rather of its standard error.

16 To check whether this is the case, we first compute the propensity score (i.e. the probability of enrolling in one of the surveyed MOOCs in 2015) using the observed predetermined characteristics mentioned in Table 1. We find that the number of treated individuals falling out of the common support is only 9 in the overall sample and 1 and 15 for the ‘Business Intelligence’ and ‘Communication and Marketing’ MOOCs, respectively.

17 A total of 147 individuals who answered the pre-questionnaire in 2017 answered the short follow-up questionnaire in 2018. In order to assess the effect of replying to the follow-up questionnaire we compare the estimates of two regressions using two different control groups: (i) including all individuals answering the pre-questionnaire in 2017 (and not limiting to those with no previous experience in MOOCs); and (ii) including all individuals answering the pre-questionnaire in 2017 (and not limiting to those with no previous experience in MOOCs) and the follow-up questionnaire in 2018. They are very similar, suggesting that this concern is not a big threat to our main estimate. However, only 32 of this enlarged control group had no previous experience with MOOCs and therefore are part of our control group. If we run a regression with this very small control group the estimate is β = 0.060 (s.e. = 0.052) [p = 0.253].

18 The questionnaire assumed that responses to the wages question would be given in euros. In order to prevent measurement error issues we restrict our analysis to European residents, which substantially reduces the number of observations.

19 Results from an ordered probit also point to a null effect of MOOCs on wages. Results available upon request.

20 Factor analysis supported the division of the items into these two dimensions. The four items composing dimension 1 were participating in one or more MOOCs in the past was useful for (a) doing new things in my current job, (b) acquiring valuable skills in the labour market, (c) acquiring enhanced skills for my current job and (d) keeping me updated in my field. The four items composing dimension 2 were participating in one or more MOOCs in the past was useful for (a) getting a pay increase, (b) getting a promotion, (c) changing to a new career, and (d) changing to a better job. The response option for each item was on a Likert scale from 1 (not useful at all) to 5 (very useful). Both dimensions are reliable with Cronbach's alpha = 0.932 for dimension 1 and 0.834 for dimension 2).

21 ‘Communication and Marketing’: dimension 1, mean 3.49 (Std. Dev. = 1.09), versus dimension 2, mean 2.02 (Std. Dev. = 1.29) (t = −10.57, p < 0.0001). ‘Business Intelligence’: dimension 1, mean 3.65 (Std. Dev. = 0.93), versus dimension 2, mean 2.20 (Std. Dev. = 2.13) (t = −9.58, p < 0.0001).

22 This result seems to be mostly driven by the participants in ‘Business Intelligence’ MOOCs (beta = −3.4, p < 0.05); however, the number of observations involved is too small to be certain.
